# Targeting ERα degradation by L-Tetrahydropalmatine provides a novel strategy for breast cancer treatment

**DOI:** 10.7150/ijbs.44005

**Published:** 2020-05-18

**Authors:** Xiaohong Xia, Jinchan He, Bin Liu, Zhenlong Shao, Qiong Xu, Tumei Hu, Cuifu Yu, Xiaolin Liu, Yuning Liao, Ningning Liu, Hongbiao Huang

**Affiliations:** 1 Guangzhou Institute of Cardiovascular Disease, Guangdong Key Laboratory of Vascular Diseases, State Key Laboratory of Respiratory Disease, the Second Affiliated Hospital of Guangzhou Medical University, Guangzhou, Guangdong, 510260, China; 2 Affiliated Cancer Hospital & institute of Guangzhou Medical University, Guangzhou Municipal and Guangdong Provincial Key Laboratory of Protein Modification and Degradation, School of Basic Medical Sciences, Guangzhou, Guangdong 511436, China

**Keywords:** breast cancer, L-THP, ERα, proliferation, cell cycle

## Abstract

The incidence and mortality of breast cancer (BCa) are the highest among female cancers. There are approximate 70% BCa that are classified as estrogen receptor alpha (ERα) positive. Therefore, targeting ERα is the most significantly therapeutic schedule. However, patients with breast cancer develop resistance to ERα or estrogen (E2) antagonists such as fulvestrant and tamoxifen. In the present study, we found that L-Tetrahydropalmatine (L-THP) significantly suppressed cell proliferation in ERα^+^ BCa cells via inducing cell cycle arrest rather than apoptosis. Additionally, L-THP enhanced the sensitivity of ERα^+^ BCa cells to tamoxifen and fulvestrant. Mechanically, the application of L-THP promotes ERα degradation through accumulating ubiquitin chains on ERα. Overexpressing ERα abrogates L-THP induced-antiproliferation in ERα^+^ BCa cells. Collectively, our work indicates that L-THP may represent a potentially novel therapeutic medicine for ERα^+^ breast cancer patient.

## Introduction

The incidence and mortality of breast cancer (BCa) have been studied to be the No.1 in female all over the world, resulting in seeking some more effective therapeutic schedules is an urgent matter 1. BCa is divided into many subtypes basing on different criteria, including in tumor size, lymph nodes, molecular subtypes classification and so on 2. Among these characteristic, molecular subtype is considered to be classical and significant advance. Based on hormone receptor status, breast cancer is divided into estrogen receptor, human growth factor, progesterone receptor and triple negative tumor 3. It has been demonstrated nearly 80 years ago that there are a closed relationship between breast cancer and estrogen receptor signaling 4.

Estrogen binds to receptor and activates the estrogen receptor (ER) signaling. The ER is considered as a transcription factor of nuclear receptor and owns two subtypes, including ERα and ERβ 5. ERα is expressed in approximately 70% breast cancer and has a draving and proliferative potential in breast cancer 6. Overexpressing ERα promotes the G0/G1 to S phase transition by increasing protein expression of p21 and inhibiting Cyclin D1, which are essential factors for cell cycle progression 7, 8. Clinically, ERα expression is correlated with the prognosis of breast cancer patients and thus is regarded as an important target to endocrine therapy 9. Some inhibitors are developed to target ERα. For example, tamoxifen and fulvestrant, inhibitors of antiestrogen, have been applied for ERα positive breast cancer patients and bring a significant prognostic 10, 11. However, with the disease progression, acquired resistance to the endocrine therapy has been developed in many patients who received tamoxifen and fulvestrant treatment 12. Thus, developing alternatively new agents is necessary to win the battle against BCa in future.

Rotundine (L-Tetrahydropalmatine, L-THP) is a natural tetrahydro protoberberine isoquinoline alkaloid isolated from *stephania and corydalis* 13. Clinically, L-THP is traditional Chinese medicine for 40 years and used for the treatment of cardiovascular and a lot of pains 14, 15. Studies have been demonstrated that L-THP shows a protective role in cardio, blood vessels and neural tissues via anti-apoptosis, anti-oxidant and anti-inflammation 16-18. As previously reported, natural alkaloids have also been shown their brilliant anti-cancer activities in some human cancers, such as leukemia and prostate cancer 19, 20. The alkaloids can induce cell apoptosis and cell cycle arrest by interacting with DNA in vitro or inhibiting G2/M transition 20, 21. Considering that L-THP is an isoquinoline alkaloid, some researchers explore the potential effect against cancers. L-THP plays an antiproliferative role in breast cancer MCF-7 cell line through regulating the uptake of ^99^Tc^m^-MIBI 22. Moreover, L-THP has been indicated to enhance the sensitivity of leukemia cell to doxorubicin and in combination of L-THP and berberine triggers anti-growth in MDA-MB231 breast cancer cell 23, 24.

In this study, L-Tetrahydropalmatine, as a natural product, inhibits the growth and progression of ERα positive breast cancer cells by cell cycle arrest. Mechanistically, we demonstrate that L-THP downregulated ERα protein via increasing the ubiquitination on ERα. Moreover, the transcriptional activity of ERα and its downstream signaling pathway are significantly suppressed by L-THP. Additionally, the application of L-THP enhances the anti-cancer effects of tamoxifen and fulvestrant on ERα positive breast cancer cells. Collectively, these results indicate that L-THP as a clinically painkiller could be applied to treat breast cancer via regulating ERα degradation and transcriptional activity.

## Materials and methods

### Materials

Rotundine (S2437), tamoxifen (S1238) and fulvestrant (S1191) were obtained from Sellcekchem (Houston, TX, USA). Antibodies are as follows: anti-ERα (#8644), anti-PARP (#9532), anti-p27 (#3686), anti-Rb (#9309), anti-p-Rb (#8516), anti-CDK4 (#12790), anti-p27 (#3686), anti-Cyclin D1 (#2922), anti-Bcl-2 (#15071), anti-GAPDH (#5174), anti-ubiquitin (#3936), anti-K48-Ub (#12805). All of these antibodies above were purchased from Cell Signaling Technology (MA, USA).

### Cell culture

MCF-7, BT474, T47D, MDA-MB453 and MDA-MB468 cells were purchased from ATCC (American Type Culture Collection, Manassas, VA, USA), grown in DMEM contained with 10% FBS. MCF-7 needs add 10 ug/ml insulin. 5% CO2 and 95% air were kept at 37℃ for cell culture.

### Cell viability

MTS (catalog no. G111) was applied for cell viability analysis. This reagent was from Promega Corporation (Madison, WI, USA). As previously reported in our studies 25, 26, cells were grown in 96-well plates for 16-24 h. Adherent cells were treated with L-THP at the indicated doses for different times, followed by 20 μl MTS for 2 or 3 h.

### Edu staining

The assay was worked as we reported previously 27, 28 using cell-light EdU Apollo 567 in vitro kit (Cat number: C10310-1, RiboBio, Guangzhou, China). MCF-7 and T47D cells were plated onto chamber slides. The indicate treatment was performed. Edu reagents were incubated for the additional 2 h. According to the manufacturer's instructions, cells were fixed, then incubated with reaction buffer. DAPI for cell nucleus staining.

### Cell cycle and apoptosis

Cells were placed into 6-well plates and then performed treatment of L-THP. Cells were digested and washed with PBS. As previously describe in our studies 29, 30, for cell cycle assay, the mixture containing 500 μl PBS and 2000 μl 70% ethanol were used to fix cell for one night. The stained reagents were applied in dark for 30 min, including 50 μg/ml of PI, 0.2% of Triton-X-100 and 100 μg/ml of RNase. For cell apoptosis, 500 μl binding buffer with 5 μl Annexin V-FITC and PI was used to resuspended. The kit for apoptosis was from Keygen Company (Nanjing, China). Lastly, cells were subjected to flow cytometry.

### Cell clone formation

BCa cells were plated and exposed to the indicated treatment. The treated cells were cultured into six-well plates until colonies formation. The cells were fixed and stained with crystal violet for 15 min. The images were captured by camera.

### Western blotting and Co-IP

To prepare protein for the two assays, cells were harvested after treatment of L-THP using lysis buffer (PMSF, protein inhibitor). In our previously describe 31, The concentration of protein was measured to the same. For Co-IP, the assay was performed to explore the interaction between the target proteins. Dynabeads were incubated with indicated antibody for 16-24h. Cell lysates were then added into antibody-conjugated dynabeads, shaking incubated for 1-2h. The kit was used to wash the immunocomplexes following the manufacturer's instructions, then PBST washes for thrice. Immunoprecipitants and protein lysates were subjected to western blot. Proteins were fractionated by SDS-PAGE and then transferred to a membrane. The membranes were incubated with the indicated antibodies including primary and secondary antibodies. The protein expression was reflected using X-ray films (Kodak, Japan).

### RT-qPCR

RNA was extracted using RNAiso plus (TakaRa Biotechnology, Dalian, China).

Dual-luciferase reporter promoter. The same concentration cDNAs were measure in each sample and then quantitative PCR in triplicate was performed using SYBR Green Dye. The amplification was obtained to analysis the expression of targeted genes. GAPDH was as a normal level. The primers of genes were as follows: human ERα-F: 5′ TCTTGGACAGGAACCAGGGA 3', human ERα-R: 5′ CAGAGACTTCAGGGTGCTGG 3'; human PS2-F: 5′-TTGTGGTTTTCCTGGTGTCA-3′, human PS2-R: 5′-GCAGATCCCTGCAGAAGTGT-3; human Cyclin D1-F:5′-GCTGCGAAGTGGAAACCATC-3′, human Cyclin D1-R:5′-CCTCCTTCTGCACACATTTGAA-3′; human GAPDH-F 5′TCCCATCACCATCTTCCA3', human GAPDH-R 5′ CATCACGCCACAGTTTCC3'.

### Confocal

As previously described immunofluorescence staining were done 32, 33. In brief, the treated cells were fixed and washed with PBS twice time followed by permeabilization with 0.1% Triton X-100 for 15 min. Then primary antibody was used to incubated with cells overnight at 4 ℃, followed by second antibody for 1 h. DAPI was applied for nuclei.

### Statistical analysis

The showed data are as mean SD from three independent experiments. Unpaired Students's t test is applied where appropriate to analysis the statistical probabilities. GraphPad Prism8.0 software and SPSS 16.0 are used to perform statistical test. P value of < 0.05 is judged statistically significant.

## Results

### L-THP inhibits the growth of ERα^+^ breast cancer cell lines

**Figure 1:**
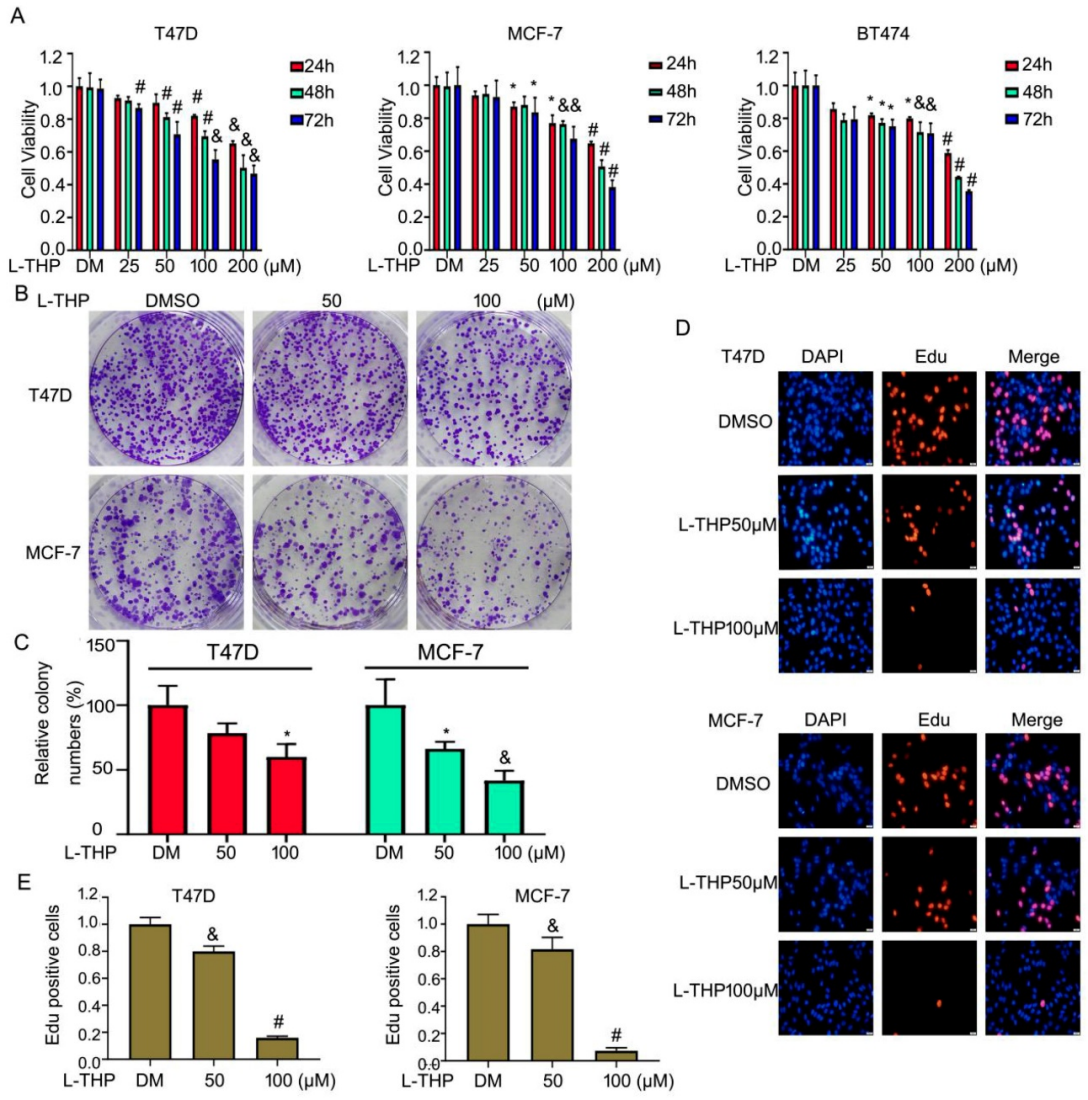
** L-THP inhibits the growth of ERα^+^ breast cancer cell lines.** (A) Cell viability was performed in MCF-7, T47D and BT474 cells post different concentrations of L-THP treatment for 24, 48 and 72h. (B) Colony formation assay was performed in BCa cells post L-THP for various concentrations for 10-14 days. (C) The colony numbers have been calculated. (D) Edu staining assay was performed in MCF-7 and T47D cells treated with L-THP for 48 h. (E) Counting stained cells which is in proliferation in each group. *p<0.05, ^&^p<0.01, ^#^p<0.001 *versus* each vehicle control. DM: DMSO.

L-THP is traditional Chinese medicine and mainly used for the treatment of cardiovascular and a lot of pains. Recently, increasing studies have shown that L-THP displays anti-proliferative effects on several cancer models, such as leukemia and ovarian cancer. But the anti-cancer activity of L-THP on ERα^+^ breast cancer cell lines has not been reported. To detect the anti-tumor effect on BCa, we choosed the three ERα^+^ breast cancer cell lines, including MCF-7, T47D and BT474 cells. Firstly, we used cell viability assay to evaluate the proliferation of BCa cells in the treatment of escalating doses of L-THP. The value of OD indicated that L-THP significantly suppressed the growth on ERα^+^ breast cancer cell lines (Figure 1A). Secondly, we also test effect of L-THP on ERα negative breast cancer cell lines (MDA-MB453 and MDA-MB468). MTS results showed that cell growth was suppressed by L-THP on ERα negative BC cells and it has been shown in Figure S1A. This clarify that L-THP may target other molecular, in addition to ERα. To further evaluate the function of ERα on inhibitory effect of L-THP on ERα^+^ breast cancer cells. We calculate the IC50 at 48 h on each cell lines. The IC50 is lower in ERα^+^ breast cancer cells than in ERα^-^ breast cancer cells (Figure S1B), suggesting ERα^+^ breast cancer cells is more sensitivity to L-THP treatment. ERα plays a significant role on L-THP's inhibitory effect. L-THP suppresses BCa cell growth depending on ERα, but just not only ERα. To determine the long effect of L-THP on BCa cell lines, colony formation assay was performed. The results showed that L-THP decreased the cloning ability of BCa (Figure 1B, C).Moreover, we further explored the cell growth using Edu staining which is a thymidine analog binding to replicated chromosomal DNA. The stained cells were decreased in the treated group of L-THP (Figure 1D, E). These findings support an inhibitory role of L-THP on ERα^+^ BCa cells.

### Anti-proliferative ability of L-THP depends on cell cycle arrest on ERα^+^ BCa cells

**Figure 2:**
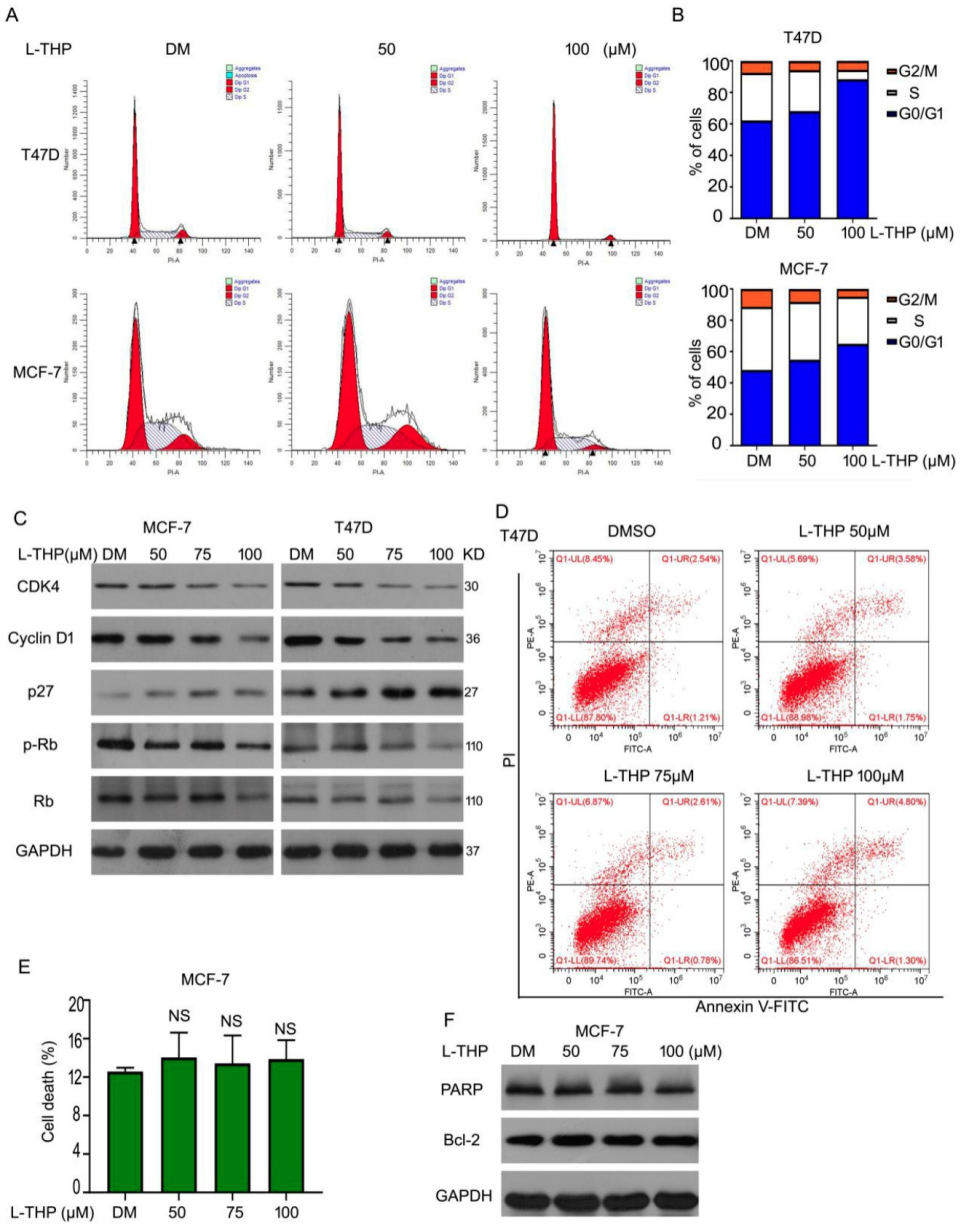
** Anti-proliferative ability of L-THP depends on cell cycle arrest on ERα^+^ BCa cells.** (A, B) The cells with L-THP treatment for 48 h were subjected to fluorescence-activated cell sorting analysis (FACS) for cell cycle distributions. (C) Cells were treated with L-THP (50, 75, 100 μM) for 48 h. And then proteins were collected for western blot assay to test the expression of CDK4, Cyclin D1, p27, Rb, p-Rb. (D) Apoptosis assay was performed on MCF-7 cells posted with L-THP treatment and (E) showed are pooled data. NS is no significant. (F) Western blot assay was performed for expression of PARP and Bcl-2.

We have demonstrated that L-THP inhibited the growth on ERα^+^ BCa cells. To study the mechanism of anti-cancer of L-THP, we considered two main aspects to explore, including cell cycle arrest and cell apoptosis. Firstly, we did the cell cycle analysis to evaluate the cell cycle distributions in present of L-THP in MCF-7 and T47D cells. The cell number was upregulated in G0/G1 phases after the treatment of L-THP, clarifying L-THP inhibited G1 to S transition in ERα^+^ BCa cells (Figure 2A, B). Furthermore, related molecular mechanisms were explored. We detected the protein expressions of p27, CDK4, Cyclin D1, Rb and p-Rb using western blotting. The expression of p27 was increased. CDK4, Cyclin D1, Rb and p-Rb were downregulated (Figure 2C). The results indicated L-THP inhibited protein expression promoting G1-S transition and upregulated protein expression suppressing transition. On the other aspect, we speculated that cell apoptosis could be inducted by L-THP. Flow cytometry assay was applied and showed no apoptotic cells in the treatment of L-THP in MCF-7 cells (Figure 2D, E). The expressions of pro-apoptotic and anti-apoptotic proteins have no change by L-THP treatment (Figure 2F). The results were consistent with the flow cytometry. Therefore, we suggest that L-THP induced inhibition of cell growth depends on cell cycle arrest rather than cell apoptosis.

### L-THP induces the downregulation of ERα protein

**Figure 3:**
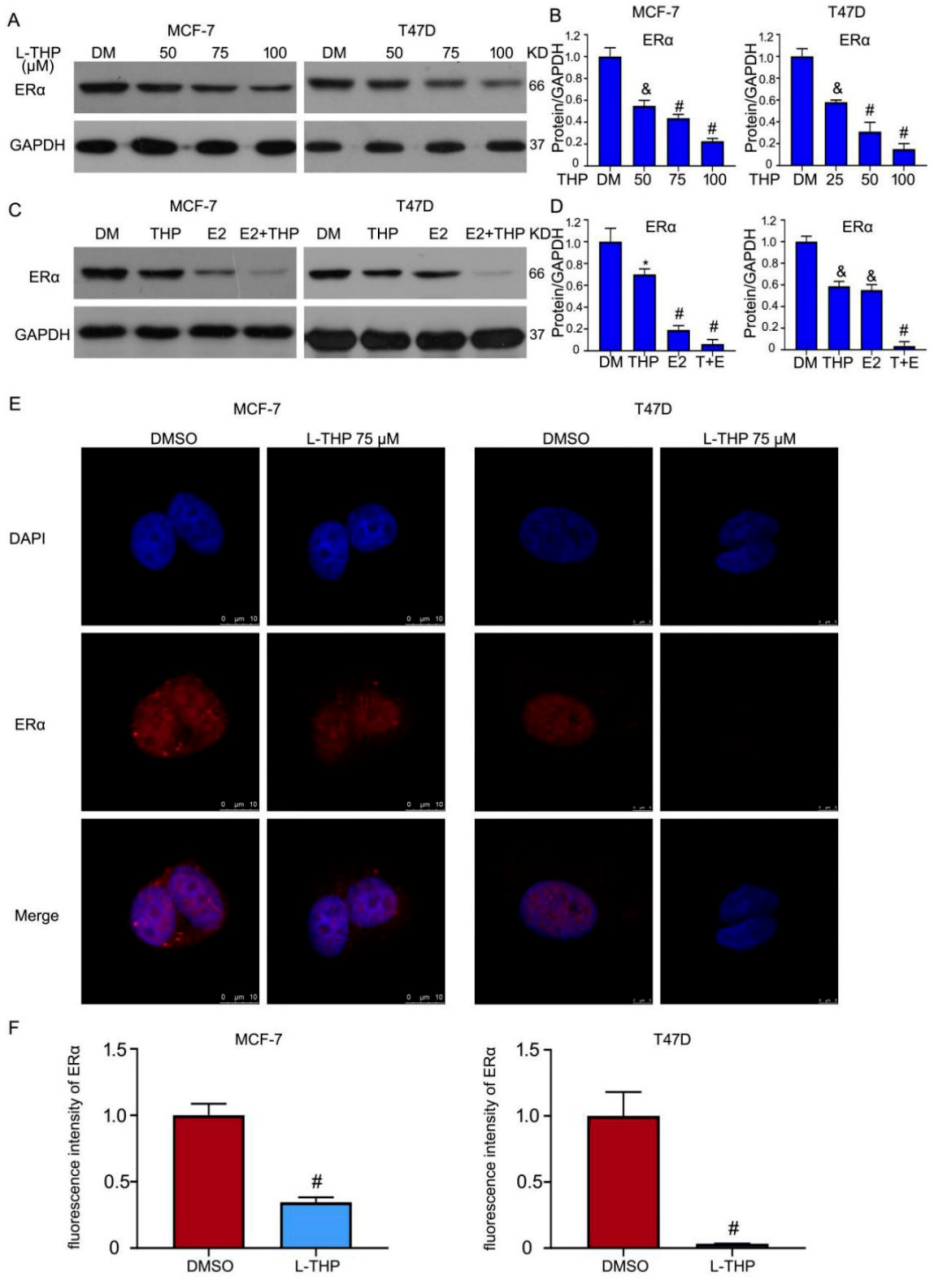
** L-THP induces the downregulation of ERα protein.** (A, C) Western blot assay was did on BCa cells posted with L-THP or estrogen (E2) treatment for 48 h. (B, D) The expressions of ERα were quantified. (E) MCF-7 and T47D cells exposed to L-THP (THP) (75 μM) for 48 h were subjected to immunofluorescence assay. Images were captured by confocal microscopy. (F) The quantification of fluorescence intensity of ERα from three images were performed. *p<0.05, ^&^p<0.01, ^#^p<0.001 *versus* each vehicle control.

Given that inhibitory effect of L-THP on ERα positive breast cancer cells, we assessed the role of L-THP on the ERα expression. Western blotting assay showed that the expression of ERα protein was decreased in MCF-7 and T47D cells (Figure 3A, B). ERα is a transcriptional factor. Estrogen (E2) can binds to ERα and then activates the transcriptional activity. E2 can promote the degradation of ERα. We sought to explore the effect of L-THP in the present of E2 on the expression of ERα protein. The decreased expression of ERα protein was more obvious induced by L-THP after adding the treatment of E2 (Figure 3C, D). Furthermore, we speculated whether L-THP can regulate co-translated in breast cancer cells. We applied the confocal microscope to observe the expression in the cytoplasm and nuclear. The images showed that L-THP significantly reduced the abundance of ERα. However, the translocate of ERα did not happen in the treatment of L-THP in MCF-7 and T47D cells (Figure 3E, F).

### L-THP decreases the expression of ERα protein resulting from promoting its degradation

**Figure 4:**
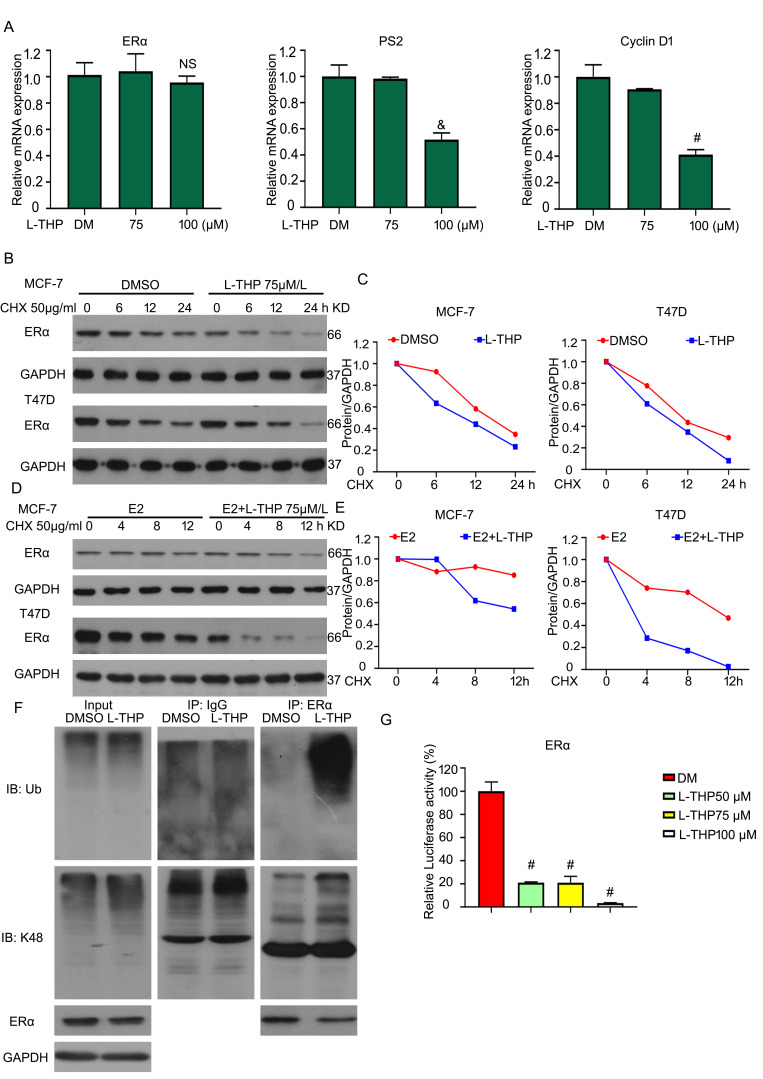
** L-THP decreases the expression of ERα protein resulting from promoting its degradation.** (A) The total RNAs were objected from T47D cells. RT-qPCR assay was performed for the expressions of ERα, Cyclin D1 and PS2 genes. (B) Proteins obtained from BCa cells exposed to CHX or CHX+L-THP treatment were subjected to western blot assay for ERα expression. (D) Western blot assay was performed on BCa cells treated with CHX+E2, CHX+E2+L-THP treatments for ERα. (C, E) The expressions of ERα were quantified. (F) BCa cells were treated with L-THP treatment for 72 h. Immunoprecipitated with ERα beads was subjected to immunoblotted for Ub and K48 expression. (G) Dual-luciferase assay was performed to detect transcriptional activity of ERα. ^&^p<0.01, ^#^p<0.001 *versus* each vehicle control.

We have demonstrated that the protein expression of ERα can be influenced by L-THP. As we all known, the protein expression is from translation and transcription methods. Next, we further explored which results in L-THP induced-decreased ERα expression between translation and transcription levels. RT-qPCR was applied to test the mRNA level of ERα. L-THP did not induce a significant change on the mRNA level of ERα, but the downstream genes of ERα were regulated by L-THP (Figure 4A). Considering the stability mRNA level of ERα, we speculated that L-THP modulated the transcription level. Cycloheximide (CHX) was used to inhibit protein synthesis. Under the treatment of CHX, L-THP accelerated the decrease of ERα protein, indicating L-THP inhibits the transcription of ERα (Figure 4B, C). In addition, in the E2 present, the downregulated speed of ERα protein was more rapid in the L-THP group (Figure 4D, E). Obtaining that L-THP inhibited the protein expression of ERα via suppressing protein synthesis, we further explored how L-THP promotes the degradation of ERα protein. Co-IP results showed that L-THP increased the poly- and K48-ubiquition levels on ERα (Figure 4F). Moreover, ERα is a transcriptional factor. The transcriptional activity of ERα was reduced by L-THP (Figure 4G).

### L-THP interacts with ERα

**Figure 5:**
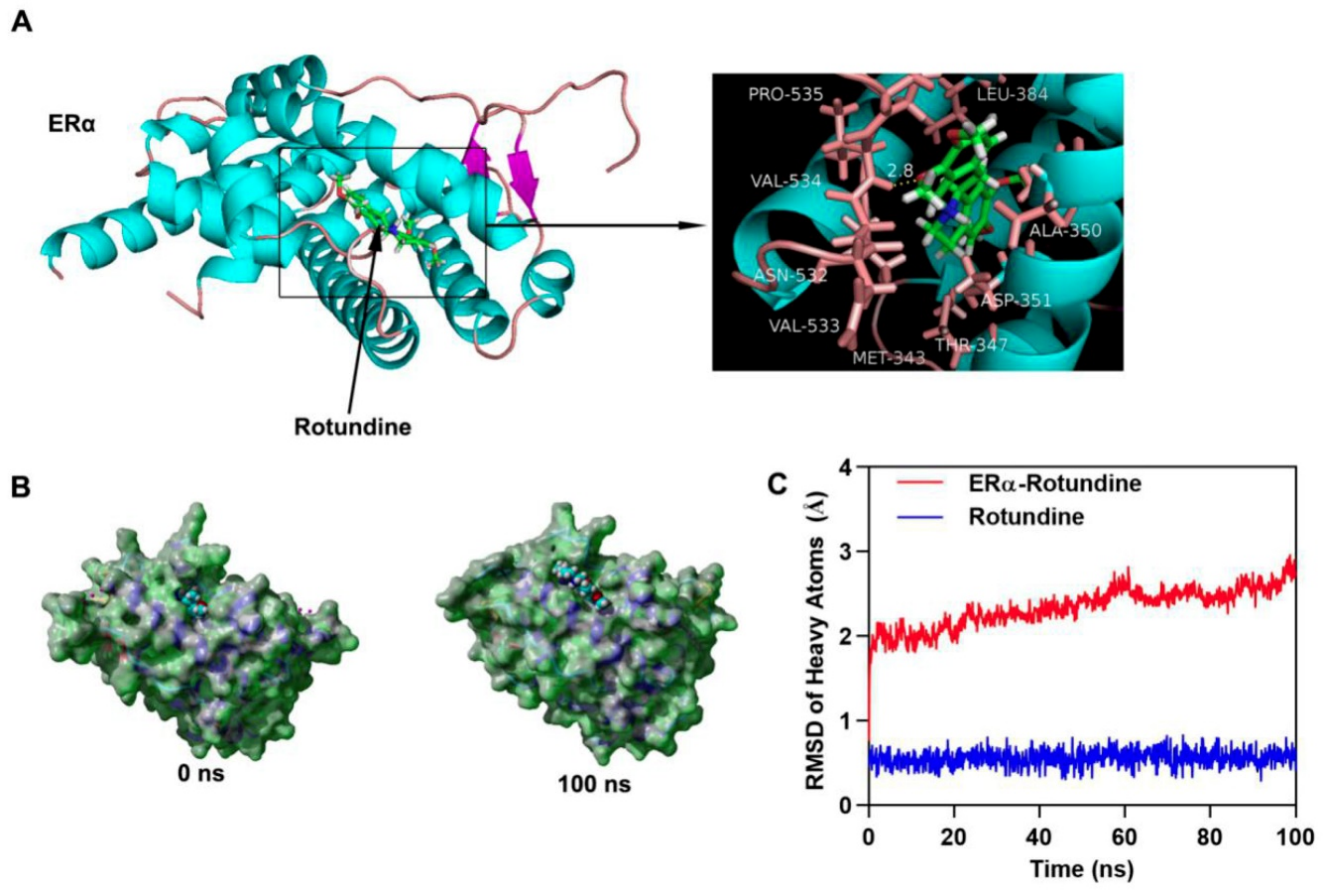
** L-THP interacts with ERα.** (A) Three dimensional crystal structure of L-THP (Rotundine) in complex with ERα (PDB ID: 5FQT). L-THP is shown in green, and the hydrogen bonds are indicated by the yellow line. (B) Surface presentation of the ERα-L-THP complex crystal structure at 0 ns and 100 ns. (C) Plots of root mean square deviation (RMSD) of heavy atoms of ERα (red) and L-THP (Rotundine) (blue) in ERα- L-THP (Rotundine) complex.

We have demonstrated the participation of L-THP on ERα protein expression. To further study how L-THP increases the ubiquition level on ERα, we observed whether exists interaction between ERα and L-THP. We performed molecular docking by using Autodock vina. The binding energy of ERα-Rotundine complex was -7.509 kcal/mol. In Figure 5A has showed that the three dimensional and two-dimensional binding conformation of ERα-Rotundine complex is observed. We found a hydrogen bond was formed between VAL-534 of ERα and L-THP. Between ERα and L-THP, the distance of hydrogen bond was detected at 2.8 Å. It was also observed that L-THP interacted with PRO-535, VAL533, ASN-532, LEU-384, ALA-350, ASP-351, THR-347, and MET-343 *via* van der Waals force. The surface models of ERα-Rotundine complex are visualized and presented (Figure 5B). L-THP steadily showed at the center of ERα binding site until the end of MD simulation. The evolution of heave atoms root-mean-square deviation (RMSD) of the complex concerning the minimized structure. The heave atoms RMSD track of ERα in ERα-L-THP complex rose from 0.6 Å to 2 Å during the first 5 ns, fluctuated around 2 Å during 5 to 20 ns, and then the RMSD rose from 2 Å to 2.5 Å during 20 to 60 ns, then fluctuated around 2.5 Å during last 40 ns (Figure 5C, red line). The heavy atoms RMSD track of Rotundine in ERα-Rotundine fluctuated around 0.6 Å during entire MD simulations (Figure 5C, blue line). These results suggest a strong binding between the kinase domain of ERα and Rotundine, indicating that Rotundine could directly target ERα.

### ERα overexpression abrogates partly growth inhibition induced by L-THP

**Figure 6:**
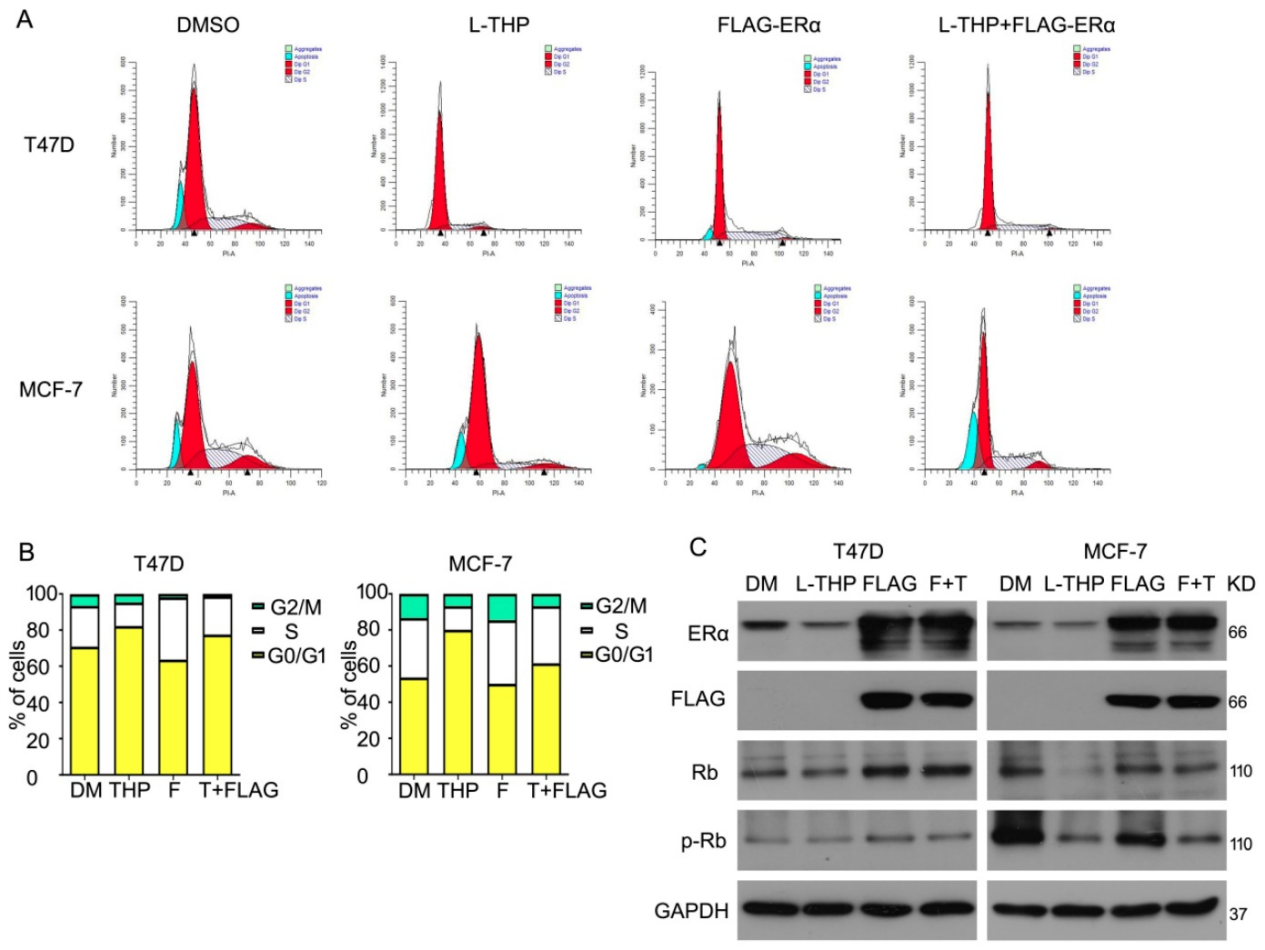
** ERα overexpression abrogates partly growth inhibition induced by L-THP.** (A) Cell cycle assay was performed on MCF-7 and T47D cells exposed to L-THP, FLAG-ERα or FLAG-ERα+L-THP. (B) Cell number was counted at different distribution. (C) Western blot assay was performed to test expression of ERα, FLAG, p-Rb and Rb proteins.

Given that L-THP inhibits cell growth and ERα expression in protein level in breast cancer cells, we lastly asked whether L-THP induced cell inhibition resulted from decreased ERα protein. To detect this idea, we purchased the plasmid of human ERα and transduced into T47D and MCF-7 cells. We found that overexpressing ERα protein promoted cell cycle progression. Importantly, under full-length plasmid of ERα treatment, cell cycle arrest induced by L-THP weaken in ERα positive breast cancer cells (Figure 6A, B). What is more, we used western blot to test the ERα and FLAG expression to ensure the plasmid into cells. The expression of two proteins were remarkably overexpressed. Rb and p-Rb expression were evaluated using western blot. The FLAG-ERα rescued the downregulation induced by L-THP. These results suggested that L-THP-inhibited breast cancer cell growth depends on ERα status (Figure 6C).

### L-THP increases the sensitivity of BCa cells to tamoxifen

**Figure 7:**
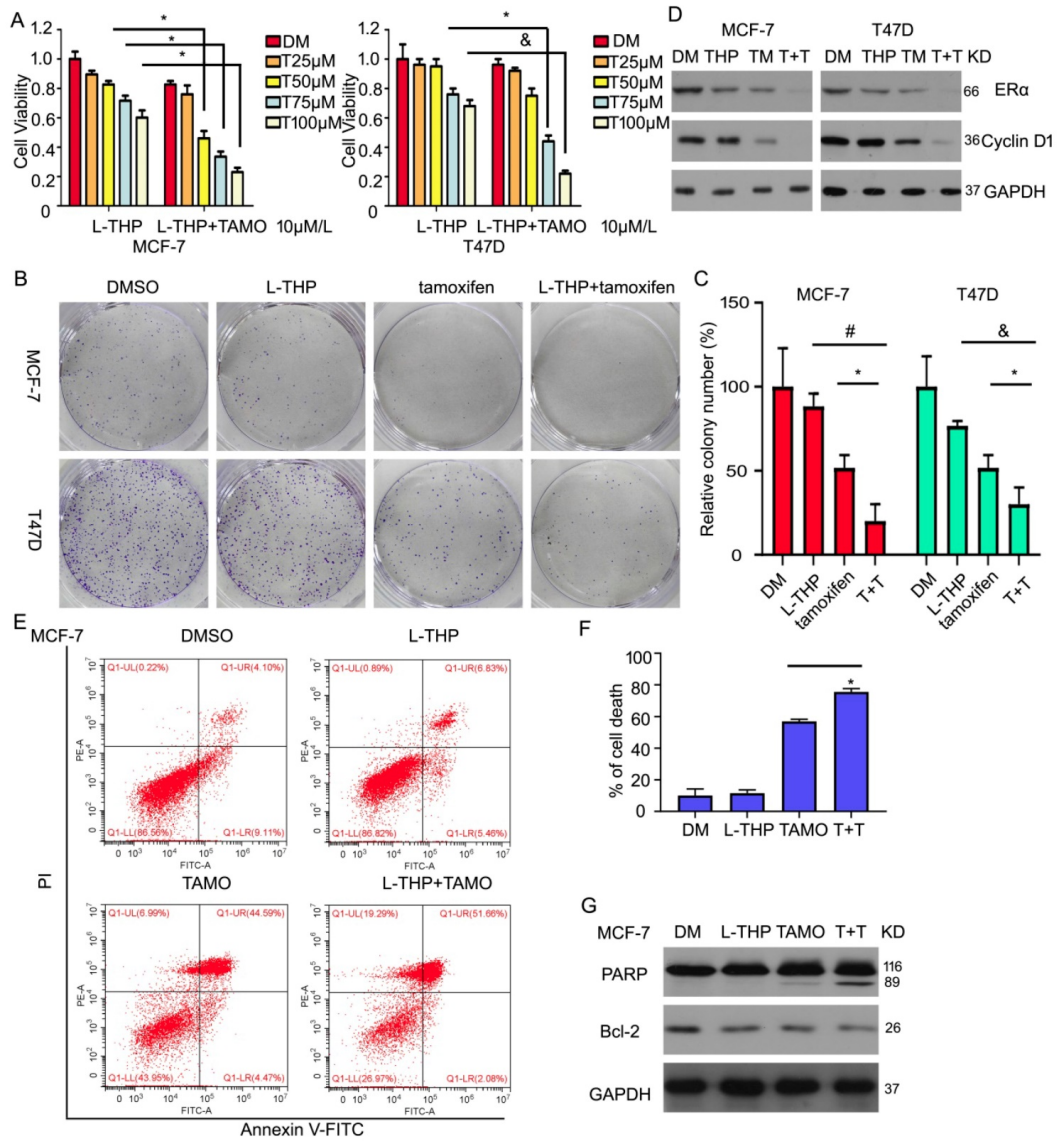
** L-THP increases the sensitivity of BCa cells to tamoxifen.** (A) Cell viability was performed on BCa cells post with L-THP at various doses or L-THP+tamoxifen (TAMO). (B) Colony formation assay was performed on MCF-7 and T47D exposed to L-THP or L-THP+tamoxifen treatment. (C) The colony numbers have been calculated. (D) Protein obtained from treated cells with L-THP or L-THP+tamoxifen was subjected to western blot assay to detect expression of ERα and Cyclin D1. (E) Cell apoptosis was performed with Annexin V-FITC staining to evaluate cell death. (F) Dead cells were counted. (G) The treated cells with L-THP or L-THP+tamoxifen was subjected to western blot for PARP and Bcl-2 expressions. *p<0.05, ^&^p<0.01, ^#^p<0.001.

Considering the role of L-THP on ERα expression and the growth in breast cancer cells, we hypothesis whether L-THP increases the effect of inhibitor targeting ERα in ERα positive BCa cells. Tamoxifen, as a antagonist of estrogen, binds to estrogen receptor. Cell viability assay were used to measure proliferation under the treatment of L-THP. We found that L-THP enhanced the inhibitory ability of tamoxifen in MCF-7 and T47D cells (Figure 7A). The long-proliferation also was suppressed by L-THP or tamoxifen. The more anti-proliferation was happened in the combination of the two drugs (Figure 7B, C). Additionally, we explore the molecular regulation, such as ERα and its targeted protein using western blotting assay. The result showed that the decreased expression of ERα and Cyclin D1 proteins were more in the group of L-THP + tamoxifen than the signal treatment (Figure 7D). In previous finding, we have demonstrated that L-THP did not induce cell apoptosis. Hence, we seek to explore whether L-THP enhanced apoptosis induce by tamoxifen in ERα BCa cells. Flow cytometry and western blotting assay clarified that cell apoptosis was increased by adding L-THP treatment (Figure 7E-G).

### L-THP and fulvestrant synergistically suppress the growth of BCa cells

**Figure 8:**
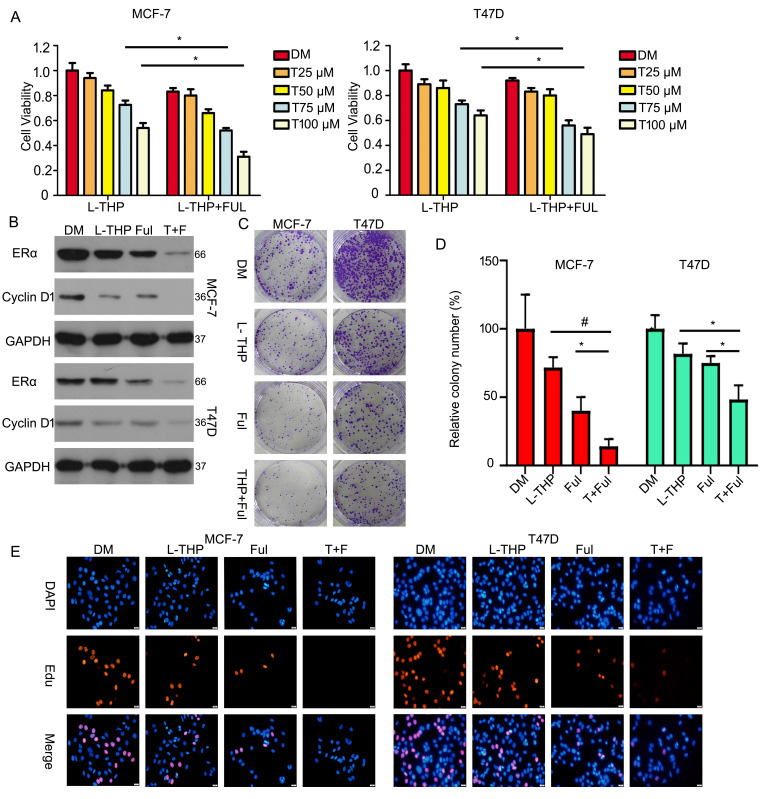
** L-THP and fulvestrant synergistically suppresss the growth of BCa cells.** (A) Cell viability was performed on BCa cells post with L-THP at various doses or L-THP+fulvestrant (FUL). (B) Protein obtained from treated cells with L-THP or L-THP+fulvestrant (FUL) was subjected to western blot assay to detect expression of ERα and Cyclin D1. (C) Colony formation assay was performed on MCF-7 and T47D exposed to L-THP or L-THP+fulvestrant (FUL) treatment. (D) The colony numbers have been calculated. (E) Edu staining assay was performed on BCa cells treated with L-THP, fulvestrant or L-THP+fulvestrant. *p<0.05, ^#^p<0.001.

Unlike tamoxifen, Fulvestrant is an antagonist of estrogen receptor that blocking the binding to estrogen to inhibit its receptor and changing receptor morphological to decrease its concentration. As the same, to better investigate the effect of L-THP on ERα protein, we test the joint effect between L-THP and fulvestrant in ERα positive breast cancer cells. We used MTS assay to evaluate cell growth. We found that L-THP enhanced the anti-proliferative ability of fulvestrant in breast cancer cells (Figure 8A). The expression of ERα and Cyclin D1 proteins were reduced when L-THP in combination with fulvestrant than the signal treatment (Figure 8B). In addition, the colony formation results also showed that effect of the combination of L-THP and fulvestrant was more significant (Figure 8C, D). We have performed the Edu staining assay and found that L-THP enhanced the sensitivity of ERα positive BCa cells to fulvestrant (Figure 8E).

## Discussion

L-THP has been used for analgesic and sedative effect in clinical practice in China for more than 40 years. L-THP belongs to tetrahydro protoberberine isoquinoline alkaloid and is naturally extracted from Corydalis and genera Stephania 34. To overcome the dose-induced toxicity of chemotherapeutic reagents in the recent year, growing studies explored the potential function of a number of natural products with less toxicity and higher potency as chemotherapeutic drugs in cancer 35. Considering that L-THP is a natural product and applied clinical therapy for pain, we ensure its safety. Some published reports claimed that L-THP owns anticancer ability. L-THP enhances the sensitivity of leukemia to doxorubicin. Moreover, L-THP in combination with berberine induces cell growth-inhibition in MDA-MB231 cell 23, 24. In this study, we discovered that L-THP suppressed cell proliferation in ERα positive breast cancer (ERα^+^ BCa).

Estrogen receptor alpha (ERα) plays a main driver in progression of breast cancer, resulting in breast cancer is classified as ERα positive breast cancer and others. ERα^+^ BCa is sensitivity to ERα inhibitor or estrogen (E2) antagonist which blocks estrogen-ERα binding. The transcriptional activity of ERα triggers multiple estrogen-responsive genes which is essential in development and survival of BCa cells. Tamoxifen and fulvestrant are regarded as important drugs for ERα positive breast cancer patients. Unfortunately, resistance to chemotherapy drugs happens in the patients with breast cancer develop. Hence, we seek to find a safe and effective drug to replace or enhance tamoxifen/fulvestrant sensitivity to breast cancer patients to reduce resistance.

Interestingly, we clarified that L-THP suppressed the growth of ERα^+^ BCa cells depends on ERα status. Cell proliferation was inhibited by L-THP in BT474, MCF-7 and T47D ERα^+^ BCa cells. The phenomenon mainly caused by cell apoptosis and cycle arrest. We found that L-THP increased cell rate at G0/G1 phase via blocking G0/G1 to S transition. But cell apoptosis be not observed. This findings suggested that anti-proliferative effect induced by L-THP results from cell cycle arrest rather than cell apoptosis. We speculated whether L-THP enhances the sensitivity of ERα BCa cells to estrogen or estrogen receptor antagonist. Tamoxifen, as estrogen antagonist, interdicts binding between estrogen and ER. Fulvestrant is an estrogen receptor antagonist. Both in combination of L-THP and tamoxifen or fulvestrant has a significant synergistic effect.

On the other hand, we further explored the most likely molecular mechanism. We found that L-THP decreased protein expression of ERα but not changed the translocation using western blotting and confocal assay. The decreased ERα may be happened in transcription and translation levels. RT-qPCR confirmed that L-THP did not induce the decreased mRNA level of ERα but downregulated its downstream genes, including PS2 and Cyclin D1. In addition to transcription, we used CHX to inhibit protein synthesis to detect the role of L-THP on ERα expression. We found that L-THP promoted the degradation of ERα protein in the presence and absence of estrogen. Based on many reported and our previously studies 30, 36, 37, we know that ubiquitin proteasome system (UPS) involves in the degradation of ERα. The level of ubiquitin on ERα was remarkably increased by L-THP treatment, suggesting L-THP promotes the degradation of ERα via accumulating the ubiquitin on ERα. Moreover, dual-luciferase reporter assay indicated L-THP inhibitory effect on the transcriptional activity of ERα. Additionally, the conformations of ERα and L-THP can be bind in three dimensional and two-dimensional, indicating that ERα interacts withL-THP. Moreover, we assessed whether the conjugate site between L-THP and ERα locates at the hinge region K302, K303 which participates degradation by UPS. However, the conjugate site is not at K302 or K303. We think that L-THP induced-ERα degradation may be via affecting some deubiquitinase or E3 ligase and so on or via changing ERα molecular structure. Delightedly, L-THP-induced cell cycle arrest can be partly abrogated by ERα overexpression.

The current study, our data confirmed that L-THP exists an inhibitory role in the growth of ERα^+^ BCa cells via promoting the degradation of ERα and suppressing its transcriptional activity. Additionally, L-THP enhances the sensitivity of ERα^+^ BCa cells to tamoxifen and fulvestrant. Therefore, this study initiates a significant function of L-THP for cancer therapy and L-THP in combination with estrogen or estrogen receptor antagonist may provide a strategy to reduce resistance in ERα^+^ breast cancer patients.

## Supplementary Material

Supplementary figures and tables.

## References

[1] Bray F, Ferlay J, Soerjomataram I, Siegel RL, Torre LA, Jemal A (2018). Global cancer statistics 2018: GLOBOCAN estimates of incidence and mortality worldwide for 36 cancers in 185 countries. CA Cancer J Clin.

[2] Reis-Filho JS, Pusztai L (2011). Gene expression profiling in breast cancer: classification, prognostication, and prediction. Lancet.

[3] Rouzier R, Perou CM, Symmans WF, Ibrahim N, Cristofanilli M, Anderson K (2005). Breast cancer molecular subtypes respond differently to preoperative chemotherapy. Clin Cancer Res.

[4] Geschickter CF, Lewis D, Hartman CG (1934). Tumors of the breast related to the oestrin hormone. Am J Cancer.

[5] Thomas C, Gustafsson JA (2011). The different roles of ER subtypes in cancer biology and therapy. Nat Rev Cancer.

[6] Perou CM, Sorlie T, Eisen MB, van de Rijn M, Jeffrey SS, Rees CA (2000). Molecular portraits of human breast tumours. Nature.

[7] Cariou S, Donovan JC, Flanagan WM, Milic A, Bhattacharya N, Slingerland JM (2000). Down-regulation of p21WAF1/CIP1 or p27Kip1 abrogates antiestrogen-mediated cell cycle arrest in human breast cancer cells. Proc Natl Acad Sci U S A.

[8] Wong SC, Chan JK, Lee KC, Hsiao WL (2001). Differential expression of p16/p21/p27 and cyclin D1/D3, and their relationships to cell proliferation, apoptosis, and tumour progression in invasive ductal carcinoma of the breast. J Pathol.

[9] Lindstrom LS, Karlsson E, Wilking UM, Johansson U, Hartman J, Lidbrink EK (2012). Clinically used breast cancer markers such as estrogen receptor, progesterone receptor, and human epidermal growth factor receptor 2 are unstable throughout tumor progression. J Clin Oncol.

[10] Brown RJ, Davidson NE (2006). Adjuvant hormonal therapy for premenopausal women with breast cancer. Semin Oncol.

[11] Hicks C, Kumar R, Pannuti A, Miele L (2012). Integrative Analysis of Response to Tamoxifen Treatment in ER-Positive Breast Cancer Using GWAS Information and Transcription Profiling. Breast Cancer (Auckl).

[12] Musgrove EA, Sutherland RL (2009). Biological determinants of endocrine resistance in breast cancer. Nat Rev Cancer.

[13] Lin MT, Chueh FY, Hsieh MT (2001). The hypothermic effects of dl-tetrahydropalmatine in rats. Neurosci Lett.

[14] Kang DW, Moon JY, Choi JG, Kang SY, Ryu Y, Park JB (2016). Antinociceptive Profile of Levo-tetrahydropalmatine in Acute and Chronic Pain Mice Models: Role of spinal sigma-1 receptor. Sci Rep.

[15] Xu SX, Yu LP, Han YR, Chen Y, Jin GZ (1989). Effects of tetrahydroprotoberberines on dopamine receptor subtypes in brain. Zhongguo Yao Li Xue Bao.

[16] Wang X, Zhao R, Zhang H, Zhou M, Zhang M, Qiao T (2018). Levo-Tetrahydropalmatine Attenuates Progression of Abdominal Aortic Aneurysm in an Elastase Perfusion Rat Model via Suppression of Matrix Metalloproteinase and Monocyte Chemotactic Protein-1. Med Sci Monit.

[17] Wu L, Ling H, Li L, Jiang L, He M (2007). Beneficial effects of the extract from Corydalis yanhusuo in rats with heart failure following myocardial infarction. J Pharm Pharmacol.

[18] Zhang MY, Liu YP, Zhang LY, Yue DM, Qi DY, Liu GJ (2015). Levo-Tetrahydropalmatine Attenuates Bone Cancer Pain by Inhibiting Microglial Cells Activation. Mediators Inflamm.

[19] Kuo CL, Chou CC, Yung BY (1995). Berberine complexes with DNA in the berberine-induced apoptosis in human leukemic HL-60 cells. Cancer Lett.

[20] Liao Y, Liu Y, Xia X, Shao Z Huang, C et al (2020). Targeting GRP78-dependent AR-V7 proteindegradation overcomes castration-resistance in prostate cancer therapy. Theranostics.

[21] Zhang X, Gu L, Li J, Shah N, He J, Yang L (2010). Degradation of MDM2 by the interaction between berberine and DAXX leads to potent apoptosis in MDM2-overexpressing cancer cells. Cancer Res.

[22] Zhao Y, Liang A, Zhang Y, Li C, Yi Y, Nilsen OG (2016). Impact of Tetrahydropalmatine on the Pharmacokinetics of Probe Drugs for CYP1A2, 2D6 and 3A Isoenzymes in Beagle Dogs. Phytother Res.

[23] Li S, Chen D, Pei R, Lu Y, Zhang P, Ma J (2017). L-Tetrahydropalmatine Induces Apoptosis in EU-4 Leukemia Cells by Down-Regulating X-Linked Inhibitor of Apoptosis Protein and Increases the Sensitivity Towards Doxorubicin. Curr Mol Med.

[24] Zhao Y, Gao JL, Ji JW, Gao M, Yin QS, Qiu QL (2014). Cytotoxicity enhancement in MDA-MB-231 cells by the combination treatment of tetrahydropalmatine and berberine derived from Corydalis yanhusuo W. T. Wang. J Intercult Ethnopharmacol.

[25] Liao Y, Liu N, Hua X, Cai J, Xia X, Wang X (2017). Proteasome-associated deubiquitinase ubiquitin-specific protease 14 regulates prostate cancer proliferation by deubiquitinating and stabilizing androgen receptor. Cell Death Dis.

[26] Xia X, Liao Y, Guo Z, Li Y, Jiang L, Zhang F (2018). Targeting proteasome-associated deubiquitinases as a novel strategy for the treatment of estrogen receptor-positive breast cancer. Oncogenesis.

[27] Liao Y, Liu N, Xia X, Guo Z, Li Y, Jiang L (2019). USP10 modulates the SKP2/Bcr-Abl axis via stabilizing SKP2 in chronic myeloid leukemia. Cell Discov.

[28] Liao Y, Xia X, Liu N, Cai J, Guo Z, Li Y (2018). Growth arrest and apoptosis induction in androgen receptor-positive human breast cancer cells by inhibition of USP14-mediated androgen receptor deubiquitination. Oncogene.

[29] Liao Y, Guo Z, Xia X, Liu Y, Huang C, Jiang L (2019). Inhibition of EGFR signaling with Spautin-1 represents a novel therapeutics for prostate cancer. J Exp Clin Cancer Res.

[30] Xia X, Liao Y, Huang C, Liu Y, He J, Shao Z (2019). Deubiquitination and stabilization of estrogen receptor alpha by ubiquitin-specific protease 7 promotes breast tumorigenesis. Cancer Lett.

[31] Liu N, Guo Z, Xia X, Liao Y, Zhang F, Huang C (2019). Auranofin lethality to prostate cancer includes inhibition of proteasomal deubiquitinases and disrupted androgen receptor signaling. Eur J Pharmacol.

[32] Xia X, Huang C, Liao Y, Liu Y, He J, Guo Z (2019). Inhibition of USP14 enhances the sensitivity of breast cancer to enzalutamide. J Exp Clin Cancer Res.

[33] Xia X, Liu Y, Liao Y, Guo Z, Huang C, Zhang F (2019). Synergistic effects of gefitinib and thalidomide treatment on EGFR-TKI-sensitive and -resistant NSCLC. Eur J Pharmacol.

[34] Yu Q, Liu T, Li S, Feng J, Wu L, Wang W (2018). The Protective Effects of Levo-Tetrahydropalmatine on ConA-Induced Liver Injury Are via TRAF6/JNK Signaling. Mediators Inflamm.

[35] Teijaro CN, Adhikari A, Shen B (2019). Challenges and opportunities for natural product discovery, production, and engineering in native producers versus heterologous hosts. J Ind Microbiol Biotechnol.

[36] Berry NB, Fan M, Nephew KP (2008). Estrogen receptor-alpha hinge-region lysines 302 and 303 regulate receptor degradation by the proteasome. Mol Endocrinol.

[37] Xue M, Zhang K, Mu K, Xu J, Yang H, Liu Y (2019). Regulation of estrogen signaling and breast cancer proliferation by an ubiquitin ligase TRIM56. Oncogenesis.

