# Supplementary material for: Targeting ERα degradation by L-Tetrahydropalmatine provides a novel strategy for breast cancer treatment
Source: Int J Biol Sci. 2020 May 18;16(12):2192–204. doi: 10.7150/ijbs.44005 (PMC7294940; doi:10.7150/ijbs.44005)
Supplement: Supplementary file 1 — Supplementary figures and tables. [file ijbsv16p2192s1.pdf]

A

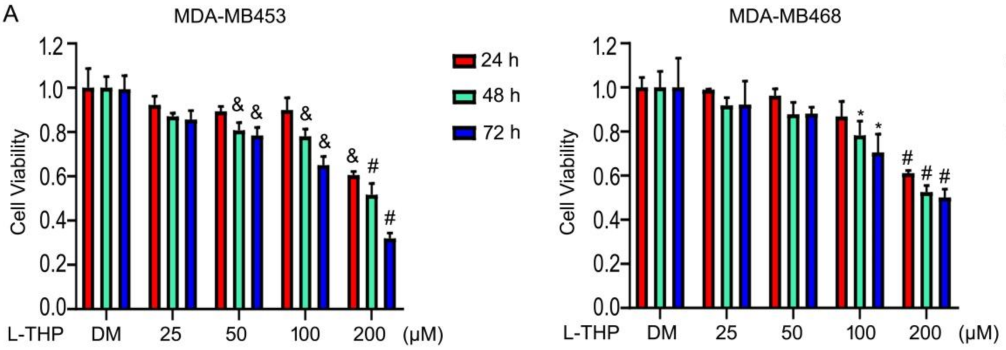

B

Cell line	T47D	MCF-7	BT474	MDA-MB453	MDA-MB468
IC50 (μM)	199.9231	203.88	177.4	207.135	214.782609

Figure S1. The inhibitory effect of L-THP on ERα^{-} breast cancer cells. (A) Cell viability was performed on MDA-MB453 and MDA-MB468 cells. (B) IC50 of L-THP on breast cancer cell lines at 48 h. $^{*}p<0.05$, $^{\&}p<0.01$, $^{\#}p<0.001$ *versus* each vehicle control. DM: DMSO.
